# IKAROS—how many feathers have you lost: mild and severe phenotypes in *IKZF1* deficiency

**DOI:** 10.3389/fped.2024.1345730

**Published:** 2024-05-09

**Authors:** Timmy Strauss, Julia Körholz, Hye Sun Kuehn, Agustin A. Gil Silva, Franziska Taube, Karolin Trautmann-Grill, Anna Stittrich, Leonora Pietzsch, Ralf Wiedemuth, Volker Wahn, Horst von Bernuth, Sergio D. Rosenzweig, Maria Fasshauer, Renate Krüger, Catharina Schuetz

**Affiliations:** ^1^ Department of Pediatrics, Faculty of Medicine and University Hospital Carl Gustav Carus, Technische Universität Dresden, Dresden, Germany; ^2^ University Center for Rare Diseases, Faculty of Medicine and University Hospital Carl Gustav Carus, Technische Universität Dresden, Dresden, Germany; ^3^ Faculty of Medicine and University Hospital Carl Gustav Carus, University Center for Chronic Immunodeficiencies (UCID), Technische Universität Dresden, Dresden, Germany; ^4^ Immunology Service, Department of Laboratory Medicine, NIH Clinical Center, Bethesda, MD, United States; ^5^ Department of Hematology and Oncology, Faculty of Medicine and University Hospital Carl Gustav Carus, Technische Universität Dresden, Dresden, Germany; ^6^ Department of Human Genetics, Labor Berlin Charité-Vivantes GmbH, Berlin, Germany; ^7^ Department of Pediatric Respiratory Medicine, Immunology, and Critical Care Medicine, Charité–Universitätsmedizin Berlin, Freie Universität Berlin, Humboldt-Universität zu Berlin, Berlin Institute of Health, Berlin, Germany; ^8^ Berlin Institute of Health (BIH), Charité—Universitätsmedizin Berlin, Berlin, Germany; ^9^ Berlin-Brandenburg Center for Regenerative Therapies (BCRT), Charité—Universitätsmedizin Berlin, Freie Universität Berlin, Humboldt-Universität zu Berlin, and Berlin Institute of Health (BIH), Berlin, Germany; ^10^ ImmunoDeficiencyCenter Leipzig (IDCL), Hospital St. Georg GGmbH Leipzig, Academic Teaching Hospital of the University of Leipzig, Leipzig, Germany

**Keywords:** *IKZF1*, IKAROS, transcription factor, haploinsufficiency, immunodeficiency

## Abstract

Heterozygous germline variants in human *IKZF1* encoding for IKAROS define an inborn error of immunity with immunodeficiency, immune dysregulation and risk of malignancy with a broad phenotypic spectrum. Growing evidence of underlying pathophysiological genotype-phenotype correlations helps to improve our understanding of IKAROS-associated diseases. We describe 6 patients from 4 kindreds with two novel *IKZF1* variants leading to haploinsufficiency from 3 centers in Germany. We also provide an overview of first symptoms to a final diagnosis including data from the literature.

## 1 Introduction

The protein IKAROS, encoded by *IKZF1*, is part of the zinc finger transcription factor family which plays a central role in lymphocyte, erythroid, myeloid and megakaryocyte differentiation and development (1–3). Germline heterozygous variants in human *IKFZ1* result in inborn errors of immunity (IEI) with infection susceptibility, immune dysregulation and risk of malignancy. The broad clinical spectrum includes bacterial, viral or fungal infections, autoimmunity, atopy, lymphoproliferative disorders and hematologic malignancies (4–7). To date, 4 different mechanisms impairing the function of IKAROS proteins have been described: haploinsufficiency (HI), dimerization defect (DD), dominant negative (DN) and gain-of-function (GOF) (8). Interestingly, regardless of the affected domain (and its corresponding functional defect), patients with IKAROS-associated diseases present with a phenotype of increased infection susceptibility and signs of immune dysregulation. Most patients with IKAROS **haploinsufficiency (HI)** suffer from bacterial infections and symptoms of immune dysregulation (8). Around 5% of patients develop malignancies including B-cell acute leukemia (B-ALL) (9). The median age of symptom onset is 10 years but first symptoms may present up to the age of 60 (10, 11). Some carriers may be clinically asymptomatic (12–14). Immunological characterization of IKAROS-HI usually reveals an incomplete B-cell arrest with low B-cell numbers and serum immunoglobulin levels. Reported patients were treated with corticosteroids, immunoglobulin replacement therapy (IgRT), prophylactic and/or therapeutic antibiotics and hematopoietic stem cell transplantation (HSCT) depending on the severity of their clinical presentation (8).

Variants located in the dimerization domain or nonsense variants upstream of the dimerization domain so called “**dimerization defect**” (DD), are usually characterized by hypogammaglobulinemia and hematological manifestations, including autoimmune cytopenias, lymphoproliferative disorders and a variety of hematologic malignancies. In comparison to patients with HI variants, those with DD variants show moderate B-cell lymphopenia and hypogammaglobulinemia resulting in less frequent and severe bacterial infections (5). Most children manifest before the age of 10 and are treated with immunosuppressants and IgRT (10).

Patients with **dominant negative (DN)** *IKZF1* variants present with severe and invasive infections [esp. *Pneumocystis jirovecii* pneumonia (PCP)], usually before 2 years of age. Immunologically they are characterized by a combined immunodeficiency (CID) with agammaglobulinemia, severe B-cell lymphopenia, absence of plasma cells and an abnormal T-cell compartment with increased naïve and reduced memory cells. Patients are often managed with antimicrobial therapy including PCP prophylaxis, IgRT, and, due to the severity of the disease, HSCT (4, 15).

While HI, DD and DN variants cause an IKAROS loss-of-function (LOF), **gain-of-function variants** in *IKZF1* lead to an increased IKAROS DNA binding (16). The clinical phenotype is dominated by signs of immune dysregulation and atopy. Multiple autoimmune phenomena like gastrointestinal, endocrinological and hematologic manifestations have been reported. Individuals with GOF variants manifest with incomplete penetrance between 1 and 40 years of age and exhibit normal B-cell numbers and normal to slightly elevated immunoglobulin levels.

**Figure 1:**
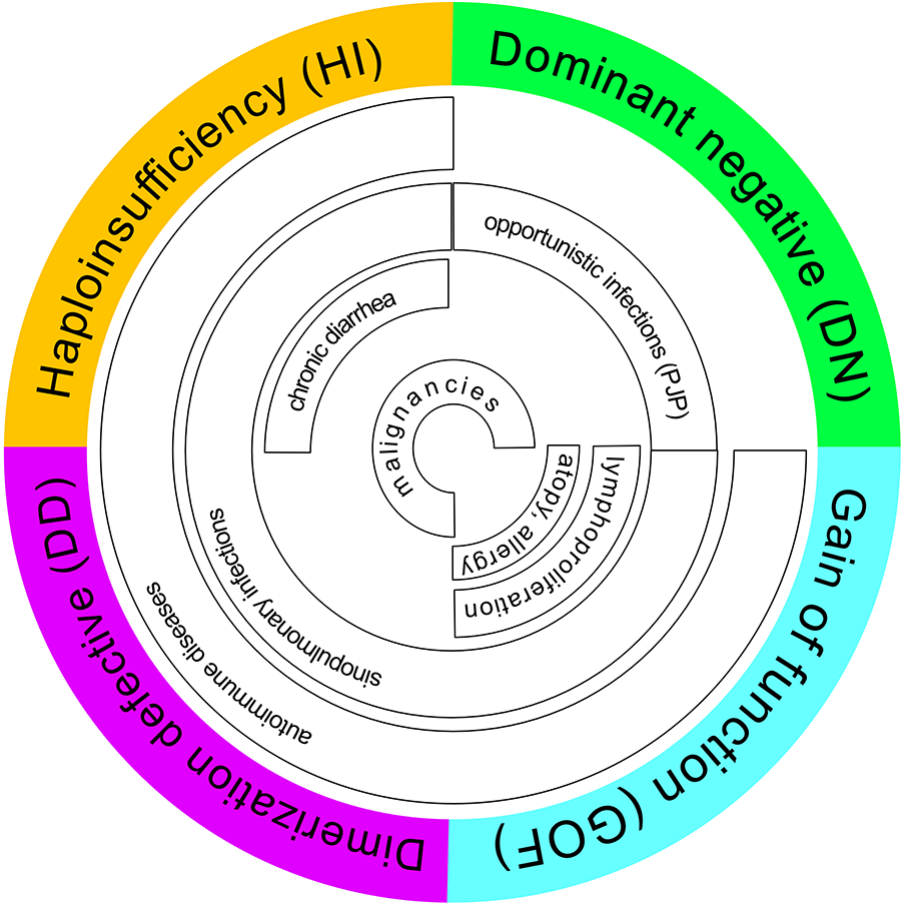
Phenotype-genotype correlation including overlapping features (review of the literature). PCP, pneumocystis jirovecii pneumonia.

In summary, diagnosis and treatment of IKAROS-associated phenotypes can be challenging due to the broad spectrum of clinical signs and symptoms (Figure 1). We subsequently describe 6 affected patients from 4 kindreds with *IKZF1* variants and functional IKAROS-HI, and provide a clinical overview from first symptoms to final diagnosis.

## 2 Clinical vignettes and functional testing

### 2.1 Family A

**Figure 2:**
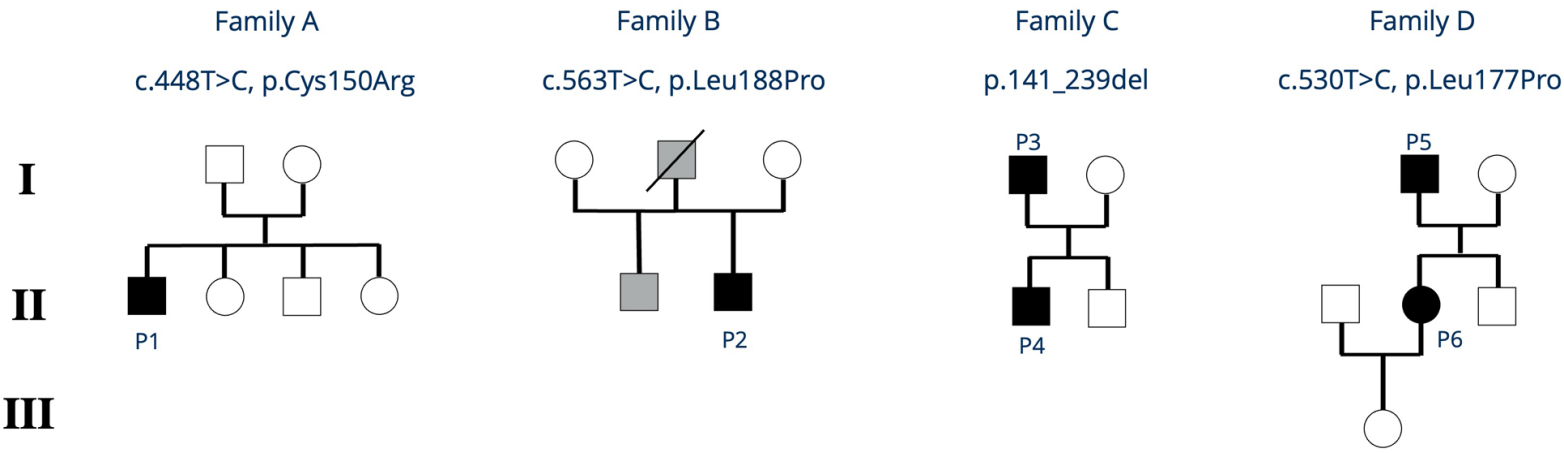
Family trees of all reported families with *IKZF1* HI. Black symbols indicate clinically affected index patients, grey symbols indicate individuals who are clinically affected but not genetically tested. P, patient.

Patient 1 is a 14-year-old boy presenting with osteomyelitis and streptococcal bacteremia at age 7. He has a history of recurrent otitis media, chronic purulent rhinitis and cough for 2 years as well as recurrent and long-lasting infections starting in childhood. His family reported frequent exhaustion upon physical activity. Between the age of 6 and 9 years, the child had intermittent diarrhea, without any causative pathogen ever detected. Intolerance for fructose or lactose was excluded, endoscopic investigation was not performed. Imaging at age 9 showed mild bronchiectasis and hepatomegaly. Further diagnostic workup revealed severe hypogammaglobulinemia and a lack of vaccination responses to tetanus, diphtheria and *Haemophilus influen*zae. Further immunologic workup showed severely reduced B cells with a predominance of naïve B cells, absent class-switch, a reduced CD4/CD8-T-cell ratio due to expanded CD8+ T cells, as well as severely reduced naïve T cells (summarized in Table 1, details in Supplementary Table S1). T-cell proliferation *in vitro* was reduced to specific antigens such as tetanus and cytomegalovirus (CMV) (positive tetanus vaccination status, unknown status of CMV exposure). A *de novo IKZF1* missense variant [c.448T>C; p.(Cys150Arg)] was identified in whole exome sequencing (WES) (Figure 2). Considering the cumulative infectious burden with possible organ damage and risk for hematological malignancies led us to reconsider curative treatment options. HSCT from an HLA-identical sister was performed at 11.5 years of age following myeloablative conditioning with treosulfan, fludarabine and thiotepa. Three years post HSCT, the patient is well with full donor chimerism including a normal T- and B-cell compartment.

**Table 1:** Summary of clinical, immunological and genetic characteristics of *IKZF1* HI and treatment of the 6 affected individuals.

	Age at symptom onset	First point of contact	Clinical symptoms	Immunological workup	Management	*IKZF1* variant
Patient 1	7	Infectious diseases	- Osteomyelitis - Streptococcal bacteriemia - Recurrent upper airway infections - Chronic diarrhea	Lymphocyte count = B-cell count ↓ CD4/CD8 ratio ↓ Immunoglobulins ↓	IgRT, HSCT	c.448T>C, p.Cys150Arg (ACMG class 4, likely pathogenic)
Patient 2	2	Hematology	- Bleeding diathesis - Chronic immune thrombocytopenia	Lymphocyte count ↓ B-cell count ↓ CD4/CD8 ratio ↓ Immunoglobulins↓	Prednisolone, IgRT, Thrombopoietin-receptor-agonists	c.563T>C, p.Leu188Pro (ACMG class 3, uncertain)
Patient 3	18	Immunology	- Recurrent upper airway infections - Esophageal strictures - Epilepsy - Sepsis, pulmonary failure requiring ECMO	Lymphocyte count = B-cell count ↓↓ CD4/CD8 ratio ↓ Immunoglobulins ↓	IgRT, cotrimoxazole	p.141_239del del Exon4 + 5
Patient 4	3	Immunology	- Recurrent upper airway infections	Lymphocyte count = B-cell count progressively ↓ CD4/CD8 ratio ↓ Immunoglobulins ↓	IgRT	p.141_239del del Exon4+5
Patient 5	55	Immunology	- Recurrent infections of upper airways - Arthritis - Eczema - Chronic diarrhea - Hypothyroidism	Lymphocyte count = B-cell count = CD4/CD8 ratio ↓ Immunoglobulins ↓	IgRT	c.530T>C, p.Leu177Pro (ACMG class 4, likely pathogenic)
Patient 6	33	Infectious diseases	- Recurrent infections of upper airways	Lymphocyte count = B-cell count = CD4/CD8 ratio ↓ Immunoglobulins =	–	c.530T>C, p.Leu177Pro (ACMG class 4, likely pathogenic)

ECMO, extracorporeal membrane oxygenation; IgRT, immunoglobulin replacement therapy; HI, haploinsufficiency.

### 2.2 Family B

Patient 2 is a 19-year-old young adult, presenting at age 2 with epistaxis and hematomas. Persistently low platelet counts, and the presence of thrombocyte auto-antibodies (anti-GPIIb/IIIa) confirmed the diagnosis of immune thrombocytopenia (ITP) treated with prednisolone and intravenous immunoglobulins. In 2023, he was started on thrombopoietin-receptor-agonists (*Revolade, NPlate*) due to recurrent episodes of symptomatic thrombocytopenia. At that time, his laboratory workup showed mild hypogammaglobulinemia, reduced IgA and IgM, and the patient was started on subcutaneous immunoglobulin (SCIG) treatment. The patient's father died from thrombosis and pulmonary embolism. The patient's half-brother (same father) also suffers from chronic ITP (Figure 2). Further immunophenotyping of the patient revealed reduced B cells, a reduced CD4/CD8 ratio due to decreased CD4+ T cells, and impaired B- and T-cell maturation with increased naïve B cells, decreased class-switched B cells, as well as reduced naïve CD4+ T cells. Vaccine responses to a variety of vaccines were undetectable (Table 1 and Supplementary Table S1). A novel heterozygous missense variant *IKZF1* [c.563T>C, p.(Leu188Pro)] was detected in WES. To date, the father and the brother have not been tested genetically.

### 2.3 Family C

Patient 3 is a 48-year-old male with a history of recurrent bacterial upper airway infections first presenting in adolescence. He reported recurrent and severe pneumonias, otitis and chronic pansinusitis (*S. aureus, H. influenzae*) requiring repeated antibiotic treatments. CT chest imaging showed bipulmonary micronodular lesions, but no bronchiectasis. At the age of 30 years, agammaglobulinemia was diagnosed. The patient was subsequently started on IgRT which improved the infection susceptibility. Of note, the patient requires 80 g IgG monthly to maintain IgG levels ≥10 g/L. Symptoms of sinusitis and productive cough further decreased when prophylaxis with cotrimoxazol was initiated. Immunophenotyping showed absent B cells, as well as a reduced CD4/CD8 ratio due to expanded CD8+ T-cells and borderline low naïve CD4+ T-cells. T cell proliferation to stimulation with mitogens and recall antigens was normal (Table 1 and Supplementary Table S2). At the age of 32 years, the patient was hospitalized with severe shingles [varicella zoster virus (VZV)]. At age 40, he started reporting recurrent episodes of diarrhea with weight loss. A previous episode of diarrhea could be explained by Salmonella infection, but the chronic intermittent course could not be attributed to any infectious agent. Histologically there were no signs of inflammation. To date the patient reports rare episodes of watery stools. In addition, he started complaining about swallowing difficulties. Endoscopy showed esophageal strictures, histology however did not confirm a suspected eosinophilic esophagitis. In his medical history, the patient reports of epilepsy with a first afebrile seizure at the age of 19, currently well-controlled with valproic acid and ethosuximide treatment. At present, the patient is obese and has both arterial hypertension and steatosis hepatis. At age 47 years, the patient was admitted to intensive care with pneumonia, pulmonary failure and fulminant sepsis. Following a very critical condition requiring extracorporeal membrane oxygenation, he has fortunately fully recovered and could be discharged from the hospital.

His now 10-year-old son (Patient 4, Figure 2) became symptomatic with recurrent upper airway infections (rhinitis, pharyngitis) starting from age 3. Due to the positive family history, clinical findings, and low IgG levels, he was started on IgRT at the age of 3 years. No major infections have occurred so far, but he also requires high doses of IgG (>0.9 g/kg body weight/month) to maintain IgG levels >6 g/L. His immunophenotyping shows progressive decline of B cells with reduced class-switch, but also a reduced CD4/CD8 T-cell ratio due to an expansion of CD8+ T-cells. Vaccination titers were low (tetanus) or undetectable (pneumococcus, measles) prior to IgRT despite respective vaccinations (summarized in Table 1, details in Supplementary Table S2).

Genetic testing of father and son revealed a deletion of exons 4 and 5 in the *IKZF1* gene of father and son.

### 2.4 Family D

Patient 5 is a now 61-year-old male with a recent history of recurrent upper airway infections starting from the age of 55. He reports 4 hospital admissions due to recurrent and prolonged pneumonias revealing mild hypogammaglobulinemia with low IgG and absent IgA levels. Chest imaging showed bronchiectasis and pulmonary granuloma. He was started on IgRT which led to reduced infectious complications. Apart from infections, he also reports arthritis in his metacarpophalangeal and proximal interphalangeal joints, eczema, hypothyroidism and several episodes of non-infectious intermittent diarrhea for over one year's duration. At present he is being re-evaluated for exacerbation of non-bloody watery diarrhea, with negative results for stool pathogens including protozoa, and normal calprotectin. He is scheduled for colonoscopy. Other co-morbidities include metabolic syndrome with arterial hypertension, coronary sclerosis, Type 2 diabetes mellitus, hepatic steatosis and sleep apnea syndrome. Immunophenotyping in this patient shows normal B-cell counts with mildly reduced class-switched B-cells, and a reduced CD4/CD8 T-cell ratio due to expanded CD8+ T-cells. Vaccination titers to tetanus toxoid and diphtheria toxoid were low prior to IgRT (summarized in Table 1, details in Supplementary Table S3).

Patient 6 is the now 34-year-old daughter of patient 5 (Figure 2). She reports long lasting respiratory infections starting in childhood without increased need for antibiotic treatment and no need for hospitalization. Her medical history is almost unremarkable without the need for prophylaxes to prevent infections. Immunophenotyping revealed normal B-cell counts, mildly impaired B-cell class-switch, mildly reduced IgA levels, normal IgG and normal vaccination titers, and increased gamma-delta T-cells (summarized in Table 1, details in Supplementary Table S3). She was found to have elevated anti-nuclear antibody (ANA)-titers 1:640 (AC-21), antimitochondrial antibody (AMA) 1:1,280 (M2 15 U/ml) so far without clinical evidence of autoimmune disease. Abdominal ultrasound revealed mild hepatosplenomegaly. Genetic testing of patient 5 and 6 revealed a novel heterozygous, pathogenic missense variant in *IKZF1* [c.530T>C, p.(Leu177Pro)].

### 2.5 Functional validation

**Figure 3:**
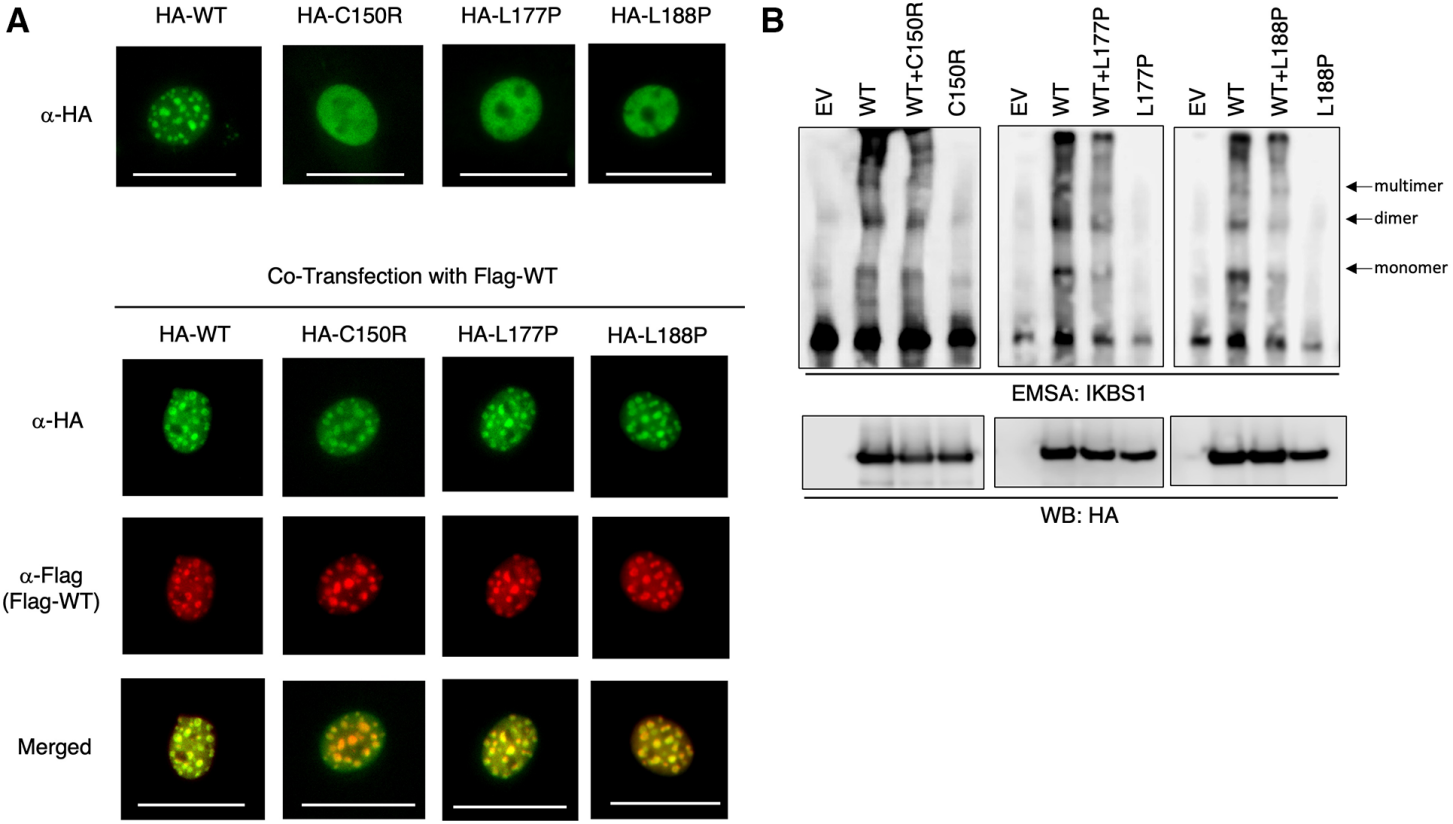
IKAROS mutants failed DNA binding and pericentromeric targeting. (**A**) NIH3T3 cells transfected with HA-tagged WT *IKZF1* and mutant *IKZF1* alone or together with Flag-tagged WT *IKZF1*. Pericentromeric heterochromatin localization of wild type and indicated mutant IKAROS proteins is revealed by fluorescence microscopy using tag specific antibodies. The scale bars indicate 25 μm. (**B**) Binding capacity of IKAROS mutants to corresponding DNA elements using an electrophoretic mobility shift assay (EMSA). Nuclear extracts of HEK293T cells transfected with HA-tagged WT *IKZF1* and/or mutant *IKZF1* were incubated with a IKBS1 DNA probe and separated by electrophoresis. Arrows indicate the monomer, dimer and multimer forms of IKAROS protein. Protein Expression of ectopic WT or mutant IKFZ1 protein was confirmed by western blot (WB) using a HA-specific antibody. EV, empty vector. Data shown are representative of three independent experiments.

Two of the herein reported four *IKZF1* heterozygous germline variants are novel. To assess their functional consequences, the 3 missense variants were subjected to *in-vitro* functional testing by immunofluorescence and electrophoretic mobility shift assay (EMSA). Immunofluorescence data showed that while NIH3T3 cells expressing WT IKAROS protein exhibit a punctate staining pattern, characteristic of pericentromeric heterochromatin localization, all three mutant proteins display diffuse nuclear staining (Figure 3A top). However, when the mutant was co-expressed with WT IKAROS protein to mimic a heterozygous condition, the normal punctate staining pattern was again observed. These data suggest that while the three mutant *IKZF1* variants lost their pericentromeric targeting, they did not exert dominant negative effects, as they did not abolish the pericentromeric localization of the WT protein under heterozygous conditions (Figure 3A bottom). Furthermore, EMSA data revealed that the ability of mutant IKAROS proteins to bind to corresponding DNA elements was completely abrogated. However, the mutants did not interfere with the binding of the WT protein under heterozygous conditions (Figure 3B). For these 3 missense variants, our data suggest that our patients with these IKAROS variants can be characterized as having IKAROS-HI. As for the patient with deletion of exons 4 and 5, the exact breaking points could not be detected and were presumably located outside of the sequenced regions. A similar deletion (chr7:50.435.843–50.452.713, 16.8 kb) was detected in a Norwegian patient with hypogammaglobulinemia and low B cells (12).

## 3 Discussion

The herein described patients all carry germline *IKZF1* variants with functional HI and have a history of recurrent bacterial or viral infections, immune thrombocytopenia and inflammatory signs like arthritis and eczema, but no history of malignant disease.

**Figure 4:**
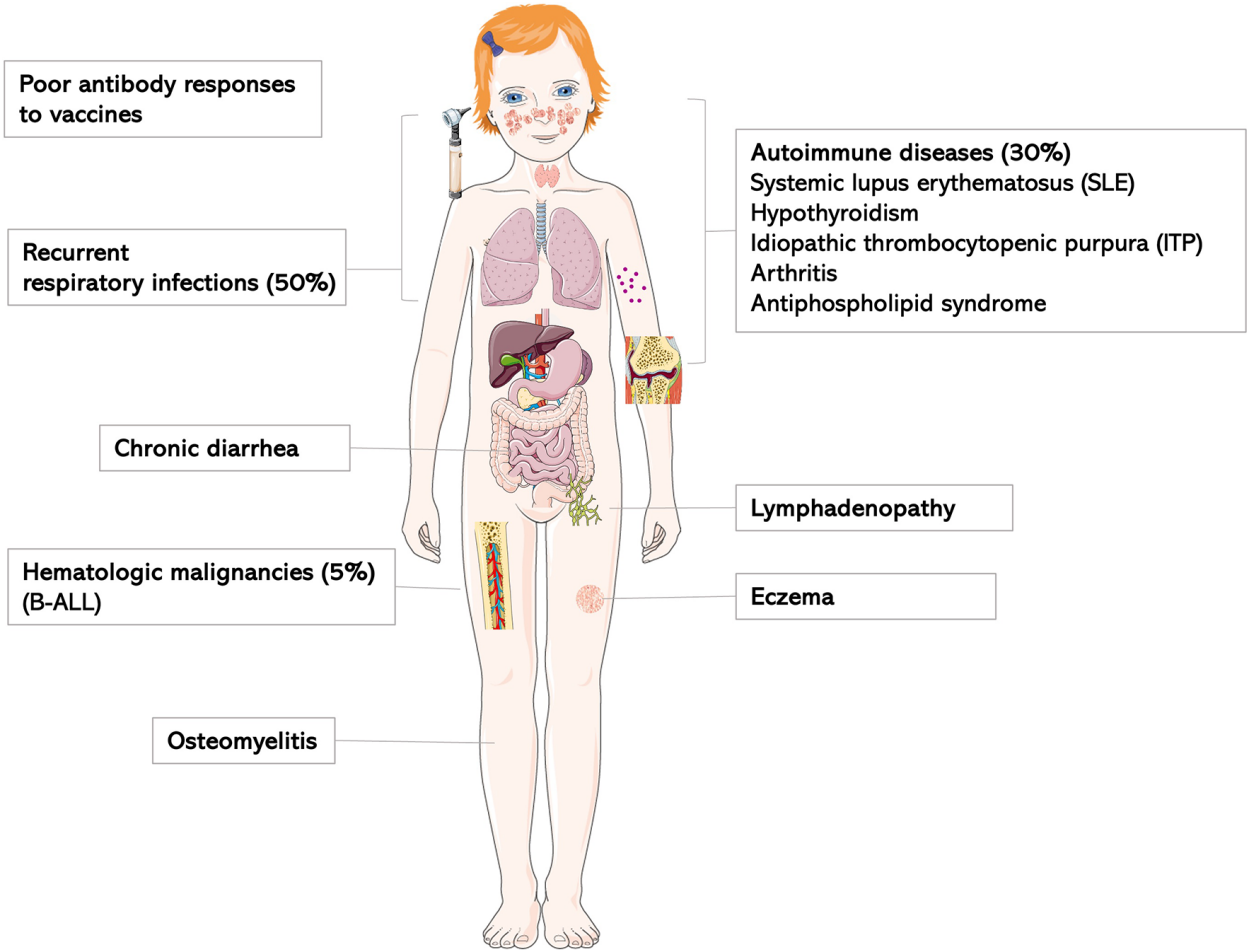
Clinical characteristics of *IKZF1*-haploinsufficiency patients as previously reviewed by PMID: 36433803.

According to previous review articles, *IKZF1* deficiency may present with a broad variety of clinical symptoms (Figures 1, 4) (5). In this IEI, a genotype-phenotype correlation has been described, i.e., signs and symptoms differ between *IKZF1* genetic variants according to their functional impact (8).

In the cohort with LOF variants presented here, the age at symptom onset ranges from early childhood until late adulthood with incomplete clinical penetrance. In agreement with the literature, the clinical spectrum spans from severe invasive infections to clinically oligosymptomatic individuals (Figure 4). Three of the 6 patients of our cohort report a history of chronic or intermittent diarrhea of infectious or undefined origin; the latter with a self-limiting course. Previous reports describe IKAROS patients with common variable immunodeficiency (CVID)-like phenotypes, of whom a minority (3 of 29) individuals had chronic or recurrent diarrhea of infectious or unknown cause (12). As in any IEI, a history of persistent or chronic diarrhea requires histological work-up based on the severity and duration of symptoms, as infectious causes may be treatable, and e.g., celiac-like disease is not a rare occurence in other IEIs, and may impact quality of life.

The immunophenotype in most of our patients showed an incomplete B-cell maturation arrest leading to a decline of B-cell numbers and serum immunoglobulin levels. These findings confirm the importance of IKAROS function in B-cell development. The most severe reduction of B-cell counts is expected in patients with dominant negative mutations, followed by haploinsufficiency and dimerization defective variants. The decline of B-cell numbers can be progressive, and the severity of B-cell deficiency may vary within one kindred (8, 14). Especially in LOF missense variants, patients additionally have abnormalities in their T-cell compartment with T-cell lymphopenia, elevated CD8+ T-cells and a decreased CD4/CD8 ratio (8). This is in line with the immunophenotyping of our patients, including one family harboring a large genetic deletion. In contrast to former reports, we also identified reduced CD4+ and CD8+ naïve T-cell numbers in some of our IKAROS-HI patients regardless of the severity of their clinical phenotype. Of note, newborns with *IKZF1* variants have been detected through severly reduced T-cell receptor excision circles (TREC) in newborn screening with severe combined immunodeficiency (SCID)-like phenotypes. Interestingly, some of these newborns had either functional T-cell abnormalities and/or partial immune-recovery on follow-up. The authors hypothesize an importance of fully functioning IKAROS during intrauterine lymphocyte development (17). Murine studies indeed confirm the importance of *IKZF1* for T-cell development (18). The biology of abnormalities in T-cell differentiation needs more scientific attention. Lastly, abnormalities of the dendritic cell (DC) compartment have also been described for IKAROS-HI patients (11). Heterozygous variants in humans have been shown to reduce peripheral DCs and to expand conventional DC1 numbers. This may indicate a regulatory effect of *IKZF1* in human DC development (19).

**Figure 5:**
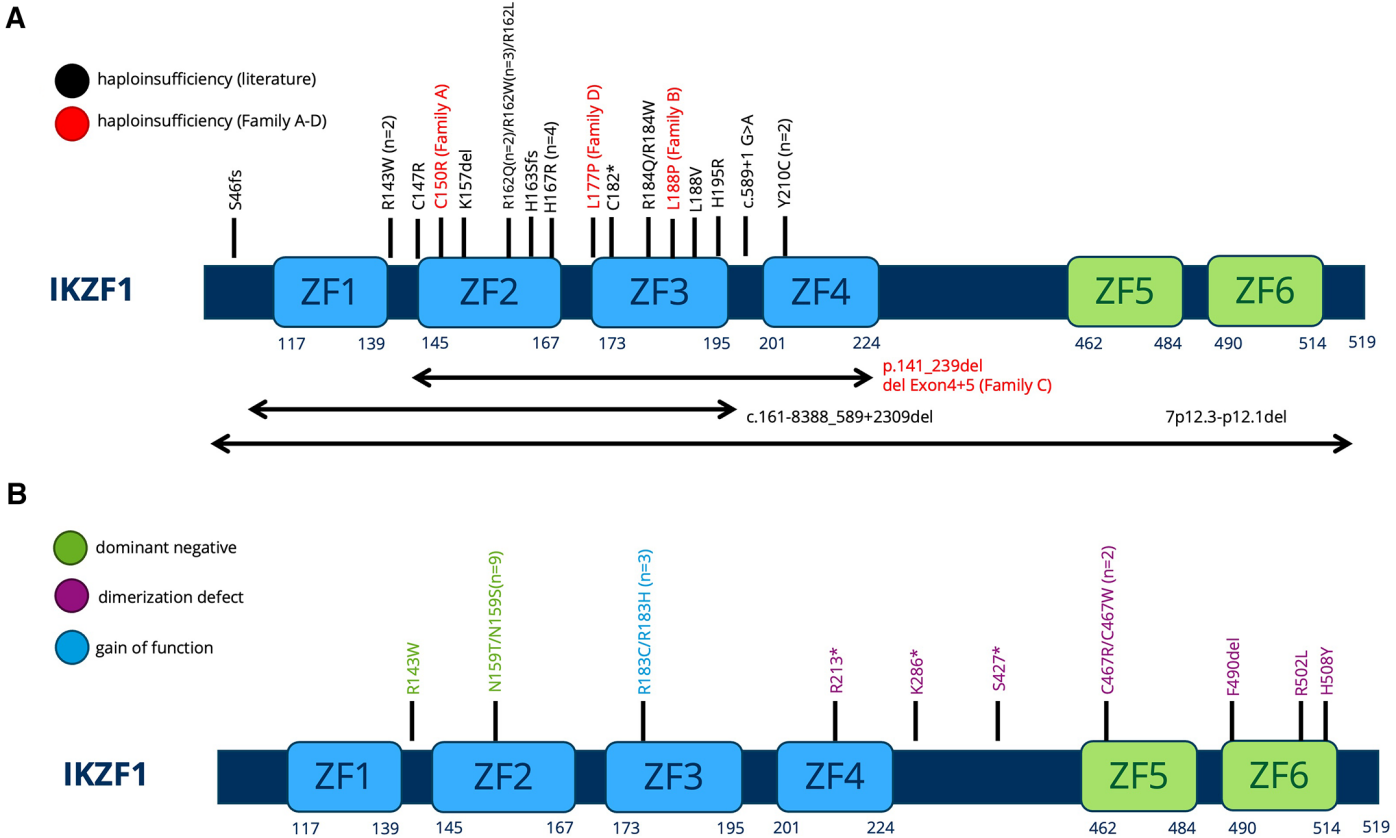
(**A,B**) Schematic of the IKAROS protein with indicated locations of the mutations of all patients previously described. The protein consists of 6 major Zinc finger (ZF) domains important for DNA binding and dimerization. (**A**) Variants leading to HI. Previously described variants are printed in black (PMID: 36433803). The families described in our manuscript are printed in red. (**B**) Dominant negative, Dimerization defect, Gain-of-function variants as previously described in PMID: 36433803.

All variants described in our manuscript—including the two novel *IKZF1* germline missense variants p.Leu188Pro (Family B) and p.Leu177Pro (Family D)—lead to the clinical phenotype of IKAROS-HI. The number of functionally proven variants is consistently growing (Figures 5A,B), with genetic heterogeneity (Figure 5A) affecting Zinc finger domains 2 and 3, which are essential for DNA binding. In our cohort, functional testing indeed confirmed the lack of DNA binding capacity and PC-HC targeting in our reported *IKZF1* variants without having dominant negative effect (Figure 3). The clinical and immunological phenotypes mostly depend on the localization of the mutation within IKAROS protein. This has been elucidated by Kuehn et al., who have dissected protein impairment due to haploinsufficiency, dominant negative, dimerization vs. GOF (8). Our cohort who has functionally validated IKAROS*-*HI, however presented with a much broader range and severity of symptoms from mild upper airway infections to severe invasive infectious complications. Therefore with the here presented cohort, we contribute to the already reported clinical spectrum of IKAROS-HI with respect to genetic variants, immunophenotype, age at manifestation, the variety of symptoms, severity of clinical presentation and therapeutic management. Nevertheless, the here reported patients show symptom overlaps with other disease phenotypes associated with *IKZF1* genetic variants such as IKAROS-DD (e.g., immune cytopenia), which is illustrated in Figure 1. This challenges a strict genotype-phenotype correlation and illustrates the importance of the interplay between genetics, clinical symptoms, functional immunological workup as well as interprofessional care to make the correct diagnosis and patient management.

Most patients reported in this manuscript have a sustained clinical response to IgRT. The patients with ITP were treated with corticosteroids and thrombopoietin-receptor-agonists. This is in in line with the successful conservative management of most IKAROS*-*HI patients in the literature: IgRT and prophylactic antibiotics for infection susceptibility, corticosteroids, high dose IgRT, rituximab for ITP and corticosteroids, immunosuppressants and anticoagulation for SLE and antiphospholipid syndrome (8, 12, 20). One of our patients received HSCT from his HLA-identical sister and shows persistent donor chimerism with cure from his IEI. This adds to the two previously reported patients with IKAROS-HI who underwent HSCT (8, 12, 20). As *IKZF1* is expressed in hematologic cells only, HSCT is a potentially curative treatment option. As in other IEI, HSCT outcomes depend on patient age and pre-transplant organ damage (21). Hence, the decision for or against HSCT should be discussed on a case-by-case basis, and be a shared decision between patients, families and treating physicians.

Alongside with the lifelong risk of severe infectious complications, one of the most decisive arguments in favour of early HSCT might be the risk of developing hematologic malignancies. *IKZF1* somatic variants are part of most oncological gene panels and a risk factor for poor outcome in hematological malignancies, mostly B-ALL (including pediatric B-cell precursor ALL) and entail more intense treatment protocols. Identification of somatic variants is relevant as these are of relevance in current protocols to guide tailoring of treatment intensity (22–24). *IKZF1* is located on chromosome 7p12.2, and comprises 8 exons. The most frequent somatic variations in B-ALL are deletions of one or more exons (25), whereas missense variants are rather rare (26, 27). Additionally, *IKZF1* copy-number variations have also been described especially in *BCR-ABL1*-positive ALL (25, 28).

Associations with hematologic malignancies are not restricted to somatic variants and have been reported in 2009 for germline *IKZF1* variants and ALL (29). The association of these variants with hematologic malignancies was revealed through identification of IKAROS-deficient patients with CVID who also developed leukemia: the here reported variants were heterozygous, and the suspected mechanism of dominance was haploinsufficiency (12). An infant diagnosed with CID and a germline *IKZF1* variant (attributed to DN IKAROS deficiency) later developed T cell leukaemia at the age of 13. In her T-ALL blasts, an additional *NOTCH1* variant could be detected (30). This further illustrates hematological malignancies on the basis of germline *IKZF1* variants, and sometimes additional somatic variants identified in hematological progenitors. Of note, hematological malignancies were reported in patients carrying variants leading to either HI, DN or DD (Figure 1).

Genetic screening of pediatric ALL cohorts, has revealed an accumulation of *IKZF1* germline variants (missense, nonsense, frameshift) (31). This raises the question if germline genetic testing should be performed in all leukemia patients, especially when they report a history of severe infections, hypogammaglobulinemia or other IKAROS-related symptoms, or in case of a positive family history (32, 33).

Pediatricians of any specialization should be vigilant when looking after patients with recurrent infections and signs of autoinflammation or autoimmunity, and/or malignancies. Taking a careful family history and initiating basic immunological workup is at the reach of most professional caretakers around the globe. In case of clinical manifestations, a combination of warning signs and/or abnormal immunophenotyping, suggestive of possible IEI genetic testing should be initiated without delay, in order not to miss variants in genes such as *IKZF1*. This may influence prophylactic and potentially curative treatment decisions like HSCT. Additionally, the risk to develop malignant disease has an impact on medical management. With this report we have provided further insight into the genotype-phenotype-correlation of IKAROS-HI and its astonishing clinical variability and lifethreatening complications, as in one individual of our cohort. The growing number of variants reported will help to broaden our understanding of gene dosage effects and understand additional mechanisms impairing IKAROS function.

## Data Availability

The raw data supporting the conclusions of this article will be made available by the authors, without undue reservation.
