# Supplementary material for: IKAROS—how many feathers have you lost: mild and severe phenotypes in IKZF1 deficiency
Source: Front Pediatr. 2024 May 9;12:1345730. doi: 10.3389/fped.2024.1345730 (PMC11135284; doi:10.3389/fped.2024.1345730)
Supplement: Supplementary file 1 [file Datasheet1.pdf]

Supplementary Material to

IKAROS - how many feathers have you lost: mild and severe phenotypes in IKZF1 deficiency

Materials and Methods

Clinical features and immunological findings

We assessed six patients from four unrelated kindreds presenting variable clinical symptoms mostly dominated by infection susceptibility and autoimmune manifestations and IKZF1 variants (see original article). The patients' main clinical and immunological characteristics are summarized in Table 1 and cases are described in more detail in section 2. Immunological investigations were performed on whole blood samples from all patients via flow cytometry during regular patient clinical care.

Identification of IKZF1 variants

Genetic testing was performed by local genetic facilities during regular patient clinical care. Whole-exome or genome sequencing was performed on whole blood DNA samples from local genetic facilities (MVZ Dresden, Labor Berlin and MedVZ Leipzig). Heterozygous germline *IKZF1* variants were identified in all patients. Written informed consent was obtained before.

Pericentromeric heterochromatin localization

NIH3T3 cells were transfected with indicated plasmids using Nucleofector kit R (Amaxa, program A-24) according to the manufacturer's instructions. After 16-24 hours, cells were washed twice with PBS and fixed with 4% paraformaldehyde for 10 minutes at room temperature. After washing cells twice with PBS, cells were permeabilized for 15 min in 0.1% Triton X-100 in PBS at room temperature and then blocked for 30 minutes in the blocking buffer (PBS with 10% FBS and 0.1% Triton X-100). Cells were stained with an anti-HA antibody (Biolegend Cat. 901501) and/or anti-Flag antibody (Cell signaling technology, Cat. 14793S). After two hours of incubation at room temperature, cells were washed with PBS and incubated with Alexa Fluor 488 (Thermo Fisher Scientific, Cat. A11001) and/or Alexa Fluor 568 -conjugated secondary antibodies (Thermo Fisher Scientific, Ca. A21069) for an hour. Cells were washed with PBS two to three times and mounted on slides using VECTASHIELD Mounting Medium (Vector Laboratories) and visualized using an EVOS M5000 cell imaging system (40X objective, Thermo Fisher scientific).

Lightshift chemiluminescent EMSA

HEK293T cells were transfected with indicated IKAROS WT and/or mutant vectors using Effectene (Qiagen) according to the manufacturer's instruction. After 48 to 72 hours incubation, nuclear extracts were prepared using NE-PER nuclear and cytoplasmic extraction kit (Thermo Fisher Scientific, Cat. 78835). Protein expressions from nuclear extracts were tested by immunoblotting. The nuclear extracts were used for the gel mobility shift assays by using LightShift Chemiluminescent EMSA kit (Thermo Fisher Scientific, Cat. 20148) according to the manufacturer's instruction. IKBS1 probe, 5′-BIOTIN- TCAGCT TTTGGGAATACCCTGTCA; reverse: 5′-BIOTIN- TGACAGGGTATTCCCAAAAGCTGA.

Supplementary table S1-3 – detailed immunological characterization of patient cohort (centers 1-3)

	Patient 1	Patient 2
Sex	**male**	**male**
Mutation	**c.448T>C, p.Cys150Arg**	**c.593T>C, p.(Leu188Pro)**
**Immunologic characterization**		
**total eosinophils (cells/µl)**	120 (0-600)	**980** (0-600)
**total monocytes (cells/µl)**	**890** (0-800)	**980** (0-800)
**total lymphocytes (cells/µl)**	2630 (1500-4000)	**1170** (1500-4000)
**CD3+ (cells/µl)**	2522 (850-3000)	1435 (850-3000)
**CD4+ (cells/µl)**	899 (450-2000)	**323** (450-2000)
**CD8+ (cells/µl)**	1331 (250-1700)	851 (250-1700)
**CD3-CD16+CD56+ (cells/µl)**	**74** (105-920)	148 (105-920)
**CD19+ (cells/µl)**	**20** (175-575)	**52** (175-575)
**naive B cells (% CD19+)**	**94.1** (52-87)	**92.2** (52-87)
**Marginal zone B cells (% CD19)**	**3.9** (4.6-18.2)	**3.3** (4.6-18.2)
**Class-swkted memory B cells (% CD19+)**	**0** (3.8-23)	**2.9** (3.8-23)
**CD21low B cells**	**0,0** (1.6-10)	**2** (1.6-10)
**CD4/CD8 ratio**	**0.68** (1-3.6)	**0.38** (1-3.6)
**CD45 RA+ (% CD4+)**	**19.9** (23-69)	**11.8** (23-69)
**CD45 RO+ (% CD4+)**	**79.2** (30-74)	**88.1** (30-74)
**Immunoglobulins**		
**IgG (g/L)**	**0.49** (7-14)	**5.6** (7-14)
**IgM (g/L)**	**< 0.05** (0.4-1.5)	**0.3** (0.4-1.5)
**IgA (g/L)**	**< 0.05** (0.7-2.3)	**0.06** (0.7-2.3)
**Vaccine response**		
**Tetanus IgG (IU/ml)**	**0.0** (>0.1)	**0.0** (>0.1)
**Diphtheria IgG (IU/ml)**	**0.0** (>0.1)	**0.0** (>0.1)
**Haemophilus influenzae IgG (U/ml)**	**0.0** (>0.15)	n.a. (>0.15)
**Pneumococcal IgG (U/mL)**	107.6 (low)	n.a.

	Patient 3	Patient 4
Sex	male	male
Mutation	p.141_239del del Exon4+5	p.141_239del del Exon4+5
**Immunologic characterization**		
**total neutrophils (cells/µl)**	6980 (1800-7970)	4210 (1800-7970)
**total eosinophils (cells/µl)**	50 (0-600)	480 (0-600)
**total monocytes (cells/µl)**	**910** (0-800)	**1110** (0-800)
**total lymphocytes (cells/µl)**	2660 (1500-4000)	2250 (1500-4000)
**CD3+ (cells/µl)**	2298 (850-3000)	**3730** (850-3000)
**CD4+ (cells/µl)**	682 (450-2000)	1190 (450-2000)
**CD8+ (cells/µl)**	1566 (250-1700)	**2320** (250-1700)
**CD3-CD16+CD56+ (cells/µl)**	202 (2016) (105-920)/ **30** (2023)	**45** (2014) (105-920)/ **8** (2023)
**CD19+ (cells/µl)**	**0** (175-575)	210 (2016) (175-575)/ **40** (2023)
**naive B cells (% CD19+)**	n.a. (52-87)	66 (52-87)
**Marginal zone B cells (% CD19)**	n.a. (4.6-18.2)	**3,6** (4.6-18.2)
**Class-swkted memory B cells (% CD19+)**	n.a. (3.8-23)	**1** (3.8-23)
**CD21low B cells**	n.a. (1.6-10)	**1** (1.6-10)
**CD4/CD8 ratio**	**0.44** (1-3.6)	**0.5** (1-3.6)
**CD45 RA+ (% CD4+)**	50 (23-69)	**75** (2016) (23-69)/ 44(2020)
**CD45 RO+ (% CD4+)**	50 (30-74)	**25** (2016) (30-74) / 54 (2020)
**Immunoglobulins**		
**IgG (g/L)**	**0.12** (7-14)	**4.72** (7-14) prior to IgRT
**IgM (g/L)**	**0.06** (0.4-1.5)	**<0.10** (0.4-1.5)
**IgA (g/L)**	**0.007** (0.7-2.3)	**0.18** (0.7-2.3)
**Vaccine response**		
**Tetanus IgG (IU/ml)**	n.a. (>0.1)	0.06 (>0.1) prior to IgRT
**Diphtheria IgG (IU/ml)**	n.a. (>0.1)	n.a.(>0.1)
**Haemophilus influenzae IgG (U/ml)**	n.a. (>0.15)	n.a. (>0.15)
**Pneumococcal IgG (mg/l)**	n.a.	< 3.3 mg/l (IgG) <1.10 mg/l (IgG2)

	Patient 5	Patient 6
Sex	**male**	**female**
Mutation	**c.530T>C, p.(Leu177Pro)**	**c.530T>C, p.(Leu177Pro)**
**Immunologic characterization**		
**total neutrophils (cells/µl)**	5410 (1800-7970)	3490 (1800-7970)
**total eosinophils (cells/µl)**	90 (0-600)	70 (0-600)
**total monocytes (cells/µl)**	**1030** (0-800)	600 (0-800)
**total lymphocytes (cells/µl)**	2390 (1500-4000)	2220 (1500-4000)
**CD3+ (cells/µl)**	**2999** (850-3000)	2443 (850-3000)
**CD4+ (cells/µl)**	727 (450-2000)	653 (450-2000)
**CD8+ (cells/µl)**	1399 (250-1700)	1248 (250-1700)
**CD3-CD16+CD56+ (cells/µl)**	290 (105-920)	**96** (105-920)
**CD19+ (cells/µl)**	194 (175-575)	257 (175-575)
**naive B cells (% CD19+)**	**152** (52-87)	**149** (52-87)
**Marginal zone B cells (% CD19)**	13,96 (4.6-18.2)	9 (4.6-18.2)
**Class-switched memory B cells(% CD19+)**	6 (3.8-23)	7 (3.8-23)
**CD21low B cells**	7 (1.6-10)	**18** (1.6-10)
**CD4/CD8 ratio**	**0.7** (1-3.6)	**0.7** (1-3.6)
**CD45 RA+ (% CD4+)**	25,7 (23-69)	37,8 (23-69)
**CD45 RO+ (% CD4+)**	70,7 (30-74)	67,4 (30-74)
**Immunoglobulins**		
**IgG (g/L)**	**5.55** (7-14)	7.9 (7-14)
**IgM (g/L)**	1.16 (0.4-1.5)	1.21 (0.4-1.5)
**IgA (g/L)**	**<0.05** (0.7-2.3)	**0.58** (0.7-2.3)
**Vaccine response**		
**Tetanus IgG (IU/ml)**	0.2 (>0.1)	>5.0 (>0.1)
**Diphtheria IgG (IU/ml)**	**<0.1** (>0.1)	0.8 (>0.1)
**Haemophilus influenzae IgG (U/ml)**	n.a. (>0.15)	n.a. (>0.15)
**Pneumococcal IgG (U/mL)**	n.a.	n.a.
